# Exploratory analysis of the human breast DNA methylation profile upon soymilk exposure

**DOI:** 10.1038/s41598-018-31767-x

**Published:** 2018-09-11

**Authors:** Louis Coussement, Selin Bolca, Wim Van Criekinge, Geert Trooskens, Klaas Mensaert, Katrien Poels, Nathalie Roche, Phillip Blondeel, Lode Godderis, Herman Depypere, Tim De Meyer

**Affiliations:** 1 Department of Data Analysis and Mathematical Modelling, Faculty of Bioscience Engineering, Ghent University, Belgium, Coupure Links 653, B-9000 Ghent, Belgium; 2 Bioinformatics Institute Ghent: from Nucleotides 2 Networks (BIG-N2N), Ghent University, Belgium, Ghent University - VIB, Technologiepark 927, B-9052 Ghent, Belgium; 3 Cancer Research Institute Ghent (CRIG), Ghent University (Hospital), Belgium, Ghent University Hospital MRB, Corneel Heymanslaan 10, B-9000 Ghent, Belgium; 4 Department of Public Health and Primary Care, Centre for Environment and Health, KU Leuven, Belgium, Kapucijnenvoer 35 blok d, box 7001, B-3000 Leuven, Belgium; 5 Department of Plastic and Reconstructive Surgery, Ghent University Hospital, Belgium, University Hospital 2K12 IC, De Pintelaan 185, B-9000 Ghent, Belgium; 6 Department of Uro-Gynaecology, Ghent University Hospital, Belgium, Corneel Heymanslaan 10, P3, B-9000 Ghent, Belgium

## Abstract

Upon soy consumption, isoflavone metabolites attain bioactive concentrations in breast tissue possibly affecting health. Though *in vitro* epigenetic activity of soy metabolites has been described, the *in vivo* impact on the epigenome is largely unknown. Therefore, in this case-control study, the breast glandular tissue DNA methylome was explored in women undergoing an aesthetic breast reduction. After a run-in phase, 10 generally healthy Belgian or Dutch women received soymilk for 5 days. MethylCap-seq methylation profiles were compared with those of 10 matched controls. Isoflavones and their microbial metabolites were quantified in urine, serum, and glandular breast tissue (liquid chromatography-mass spectrometry) and 17β-estradiol in glandular breast tissue (immunoassay). Global DNA methylation levels were obtained for 6 cases and 5 controls using liquid chromatography-mass spectrometry. Although lower MethylCap-seq coverages were observed, mass spectrometry results and computational LINE-1 methylation analysis did not provide evidence supporting global methylation alterations upon treatment. At a false discovery rate of 0.05, no differentially methylated loci were identified. Moreover, a set of previously identified loci was specifically tested, but earlier reported results could not be validated. In conclusion, after a 5-day soymilk treatment, no major general epigenetic reprogramming in breast tissue could be found in this exploratory study.

## Introduction

Epigenetic mechanisms link genotype with phenotype through reversible DNA modifications. Environmental factors, including diet, can remodel our epigenome lifelong in a beneficial or detrimental way^1^. Whether dietary exposure to epigenetically active compounds may result in any, beneficial or adverse, effect remains unclear. In particular, dietary exposure to phytoestrogens, a group of non-nutrients capable of interfering with the endogenous estrogen signaling and associated processes *in vitro* and/or *in vivo*, causes a lot of controversy and safety concerns^2–4^.

Soy is the major dietary source of the isoflavones genistein and daidzein. Upon soymilk consumption, components may reach exposure levels in human breast tissue rendering them bioactive^5^. Epidemiologic data support a small reduction in breast cancer risk, though also adverse effects have been reported^6,7^. Some proteins in soymilk, e.g. lunasin, have been attributed tumor suppressor activity, yet particularly isoflavones appear important^8^.

However, some estrogen receptor-independent mechanisms of action have been postulated, such as attenuation of steroidogenesis and metabolism, as well as regulation of gene expression through epigenetic silencing. Indeed, isoflavones, and genistein in particular, have been reported as inhibitors of DNA methyltransferases and histone deacetylases^9^. Data are mainly derived from *in vitro* experiments measuring the effect of supraphysiological concentrations on single candidate genes whereas results of *in vivo* studies on dietary isoflavone-induced epigenetic modifications and related changes in gene expression are still limited. To the best of our knowledge, the study of Qin *et al*.^10^ is the only report on the effect of dietary isoflavones on the DNA methylation degrees of a limited subset (5) of cancer-related genes in human mammary ductoscopy samples. Though no treatment-related changes were observed in this study, results suggested an association between higher post-treatment genistein levels and RARβ2 (RARB) and CCND2 hypermethylation, which might increase breast cancer risk^10^.

As epigenetic alterations in breast cancer have been related to histological and outcome data^11^, additional research is required to evaluate the *in vivo* epigenetic impact of isoflavone exposure in breast tissue, preferably in a genome-wide manner. Therefore, here, we explored the breast tissue epigenome of healthy women with and without soymilk supplementation, both globally (by liquid chromatography – mass spectrometry, LC-MS) and locally (by MethylCap-seq).

## Results

### Study population

A total of 30 healthy women undergoing an aesthetic breast reduction, all complying with the study protocol, participated in this study. However, for the methylomics analysis, 10 controls were selected based on their age and menstrual cycle or menopausal status, to match the overall characteristics of subjects in the soymilk group. As a result, the sample size was decreased to 20. The age and BMI, based on self-reported weight and height measurements, ranged from 17 to 60 y and from 18.7 to 33.0 kg/m², respectively (Table 1). Five women (25%; soymilk: 2; control: 3) were in the follicular phase of their menstrual cycle and 3 (15%; soymilk: 2; control: 1) in the luteal phase, whereas 12 (60%; soymilk: 6; control: 6) were (peri)menopausal. Two women were taking oral contraceptives (10%; soymilk: 1; control: 1). All participants reported average fat and fiber intakes, and 4 women (20%; soymilk: 2; control: 2) were smoking on a daily basis. With regard to past and present isoflavone intakes, the study population consisted of mostly non-consumers (80%), with only 2 subjects consuming soy-derived products on a daily-to-weekly basis (soymilk: 1; control: 1).

**Table 1:** Subject characteristics.

ID	Treatment	Age*	BMI	S Genistein	S Daidzein	BG Genistein	BG Daidzein	BG Total Isoflavones	BG 17β-estradiol
SB1	Soymilk	47	23.88	282.18	175.50	114.04	22.15	136.19	1.38
SB2**	Soymilk	52	29.76	1455.61	1184.52	296.24	436.25	732.49	0.45
SB3**	Soymilk	20	28.30	246.39	297.95	326.29	369.94	696.23	0.03
SB4	Soymilk	56	28.28	394.22	551.87	169.33	48.88	218.21	0.05
SB5	Control	51	21.50	0.00	0.00	0.00	0.00	0.00	0.06
SB6**	Control	18	26.60	0.00	0.00	0.00	0.00	0.00	0.88
SB7	Control	60	31.40	0.00	0.00	0.00	0.00	0.00	0.26
SB8	Soymilk	54	32.95	679.61	583.82	241.88	34.07	275.95	0.10
SB10	Control	57	22.40	0.00	0.00	0.00	0.00	0.00	0.07
SB11	Soymilk	34	24.86	353.05	816.25	194.16	51.10	245.26	0.09
SB12	Control	19	18.70	0.00	0.00	0.00	0.00	0.00	0.02
SB13	Control	52	27.70	0.00	0.00	0.00	0.00	0.00	0.20
SB14**	Control	32	25.20	0.00	0.00	0.00	0.00	0.00	0.62
SB15	Control	38	28.60	0.00	0.00	0.00	0.00	0.00	0.02
SB16	Soymilk	52	24.80	283.06	105.14	198.58	54.51	253.09	0.02
SB18	Soymilk	44	23.73	1267.21	1313.52	493.76	666.05	1159.81	1.49
SB19	Soymilk	60	25.32	2831.23	520.10	390.18	67.03	457.21	0.02
SB20	Control	56	24.20	0.00	0.00	0.00	0.00	0.00	0.04
SB21	Control	38	23.20	0.00	0.00	0.00	0.00	0.00	0.86
SB22	Soymilk	18^$^	21.48	451.08	1079.20	440.33	369.63	809.96	0.04

S and BG reflect respectively serum and breast glandular tissue samples. Note: samples SB9 and SB17 were technical replicates for MethylCap-seq analysis (see Table 2) and were therefore not listed. Units for each variable are: years, kg/m², nmol/L, and pmol/g for respectively the age, BMI, the S concentrations and the BG concentrations.

*Ages were calculated based on provided birth year and date of surgery. Age for one participant (SB22), indicated by $, was manually modified to 18, given that a rounding error led to 17 years of age, though no participants were younger than 18 years at the date of procedure.

**Smokers.

### Exposure to isoflavones and 17β-estradiol

Exposure to genistein, daidzein, and its microbial metabolites upon soy supplementation, was assessed as the sum of unconjugated aglycones and deconjugated (sulfo)glucuronides and sulfates, measured in hydrolyzed urine, serum, and glandular breast tissue. None of the urine samples collected at the end of the run-in phase after the intervention phase for the control group contained detectable amounts of isoflavones, whereas the estimated daily urinary isoflavone excretion confirmed compliance to the soymilk diet in the treatment group (genistein: 6.6–13,140.0 µmol/day; daidzein: 2.4–2,025.0 µmol/day; individual data not shown). Genistein and total daidzein (*i*.*e*., sum of daidzein, dihydrodaidzein, *O-*desmethylangolensin, and equol) serum concentrations ranged from 246.4 to 2,831 nmol/L and from 105.1 to 1,314 nmol/L upon treatment, respectively. In breast glandular tissue, exposure levels of 114.0–493.8 pmol/g genistein, of 22.2–666.0 pmol/g total daidzein, and of 0.021–1.489 pmol/g 17β-estradiol were measured. 17β-estradiol was not significantly different between soymilk and control groups (Wilcoxon rank-sum test, *P* = 0.74). Individual hormone and metabolite levels can be found in Table 1.

### DNA methylation profiles

#### Sequencing statistics and data exploration

**Figure 1:**
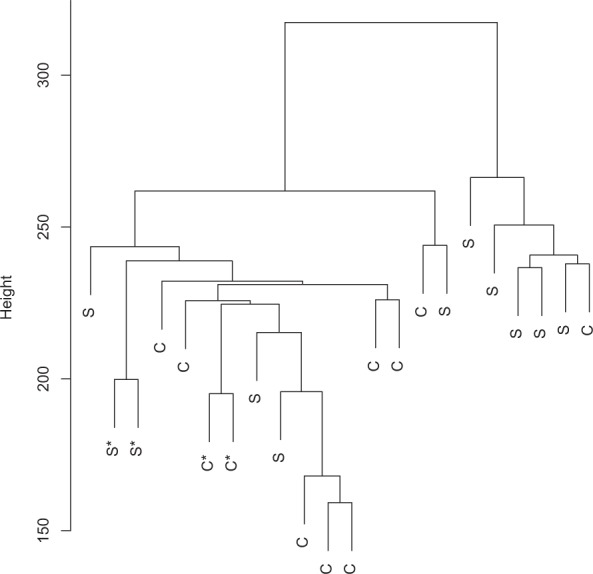
Hierarchical cluster analysis of soymilk (S) and control (C) samples based on normalized methylation values for 10000 most variable loci (complete linkage, Euclidean distances). Technical replicates are indicated by asterisks.

Overall coverages, amount and fraction of mapped fragments, and library sizes upon data summary are shown in Table 2. Hierarchical cluster analysis of soy supplementation and control samples revealed that the technical replicates cluster together (Fig. 1), yet that there is no major distinction between treated and control subjects. Though the right cluster appears to be featured by more soy-treated samples and somewhat lower coverages, no sample characteristic (age, BMI, total coverage, library size, treatment) was found to clearly explain these two clusters (data not shown).

**Table 2:** MethylCap-seq study characteristics.

ID	Treatment	Coverage	Mapped	% Mapped	Library size
SB1	Soymilk	23641310	15130395	64.00%	3230581
SB2	Soymilk	20005124	13364407	66.80%	2344832
SB3	Soymilk	20596052	13283325	64.49%	3120649
SB4	Soymilk	23605776	16486437	69.84%	2402701
SB5	Control	52472379	36240165	69.07%	4747398
SB6	Control	28784091	19727820	68.54%	2826594
SB7	Control	50403081	34620817	68.69%	4832033
SB8	Soymilk	32235327	22806551	70.75%	3336655
SB9	Control*	30410592	21433945	70.48%	NA
SB10	Control	47379658	33209046	70.09%	4353299
SB11	Soymilk	33798425	24635918	72.89%	1859969
SB12	Control	32412829	22138492	68.30%	3408381
SB13	Control	28898463	19854562	68.70%	3212742
SB14	Control	26743842	18134534	67.81%	3319438
SB15	Control	30217735	19679085	65.12%	4302979
SB16	Soymilk	21840292	14934423	68.38%	2837047
SB17	Soymilk*	25667328	18073389	70.41%	NA
SB18	Soymilk	24902261	15842246	63.62%	4044801
SB19	Soymilk	28582845	20225632	70.76%	2789297
SB20	Control	29028050	19954925	68.74%	3169451
SB21	Control*	32123694	21969096	68.39%	3446305
SB22	Soymilk*	30182885	20833618	69.02%	2894860

Columns represent id, coverage (amount of sequenced paired-end fragments), amount and fraction of mapped fragments, and final library sizes of data used for limma-voom statistical analysis. *Technical replicates, replicates with lowest coverage were not considered for statistical analysis (therefore “NA” for library size).

#### Global methylation degrees

Comparison of general sequencing characteristics between the soymilk and control groups revealed significant differences in coverage (*P* = 0.009), number of mapped fragments (*P* = 0.012) and library size upon data summary (*P* = 0.003), despite sample randomization before gDNA extraction and MethylCap-seq. The lower coverages for the soymilk samples may suggest global hypomethylation upon soy exposure. However, as reflected by the variation in sequencing characteristics for the technical replicates (Table 2), technical sequencing variation is an important, yet difficult to assess, confounder. Therefore, we attempted to find additional evidence supporting overall differences in DNA methylation between the treatment groups. These analyses were based on (i) independent measurement of methylation degree (by LC-MS) on a subset of the population under study, (ii) an evaluation of the association between the MethylCap sequencing characteristics and measured soy metabolite concentrations in the tissue for the soy-exposed group only, (iii) evaluation of LINE-1 methylation (proxy for global methylation^12^) shifts associated with soy exposure in the MethylCap-seq data (full population).

First, for a subset of 6 cases and 5 controls (i.e. 11 subjects of the total of 20), sufficient gDNA was available to measure global DNA methylation by LC-MS analysis. No significant difference was observed between cases (2.45 ± 0.55%) and controls (2.34 ± 0.46%) (mean ± standard deviation) (*P* = 0.72).

Next, if sequencing characteristics differ due to soy supplementation, one would expect that these parameters are also correlated with the *in vivo* concentrations of soy metabolites (genistein, total daidzein and total isoflavones) in soy treated individuals. However, Spearman correlation revealed no borderline or significant associations between MethylCap-seq library size and respectively genistein (ρ = 0.47, *P* = 0.18), total daidzein (ρ = 0.03, *P* = 0.95), and total isoflavone (ρ = 0.37, *P* = 0.30) tissue concentrations in those subjects exposed to soy. Additionally, the correlations are all positive, opposite of what would be anticipated if soy supplementation would lead to global hypomethylation (i.e. lower library sizes with higher isoflavone concentrations). It should be noted that the small sample size (n = 10, i.e. only cases) implies a low power.

Finally, LINE-1 repeat methylation was considered, often used as a proxy for “global methylation” levels^12^. On average, approximately 1% of all reads mapped to L1Hs, with no significant differences in LINE-1 methylation between both groups (*P* = 0.49). It should be noted that adjustment for total coverage might have adjusted for the total degree of DNA methylation as well. However, it seems unlikely that global hypomethylation equally affects all loci in the genome, implying that some (relative) methylation shifts should be observed, *cfr*. Akalin *et al*.^13^. Moreover, other relative comparisons, *e*.*g*. average genic-intergenic methylation degree ratios, promoter–genic methylation degree ratios, revealed no significant differences between treatment groups (data not shown).

#### Differential DNA methylation

**Figure 2:**
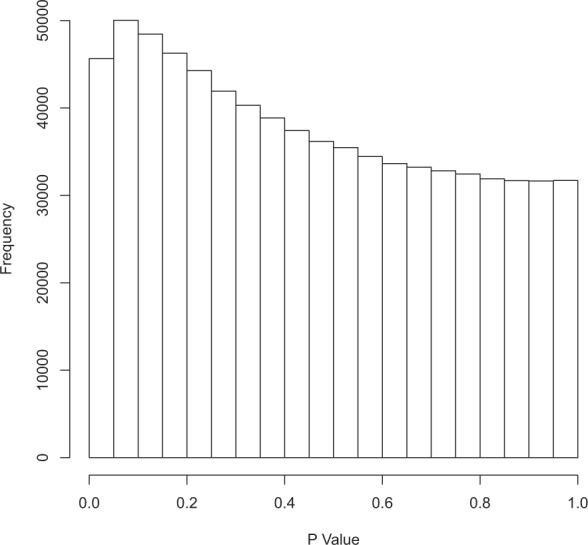
*P*-value distribution for the general differential methylation analysis.

Upon performing the extra filtering steps for minimum coverage, 758,275 variables were withheld for further analysis, leading to the final dataset (see Materials and Methods section). Given results from the global DNA methylation analysis, TMM was applied for normalization, assuming that most loci are not differentially methylated. At a false discovery rate (FDR) of 0.05, no significant loci were found using limma-voom (minimum FDR: 78,9%). The *P*-value distribution suggests that a minor treatment effect may be present in the data (minor enrichment at *P* = 0, Fig. 2), yet this may also be due to (slightly) imperfect normalization of pre-existing library size differences.

Previously, RARB and CCND2 promoter hypermethylation has been associated with *in vivo* serum genistein concentrations^10^. Moreover, as summarized by Pudenz *et al*.^14^, *in vitro* data suggests an impact of soy isoflavones on GSTP1, SCGB3A1 (HIN-1), TERT, BRCA1, BRCA2, ATM, APC, PTEN and SERPINB5 promoter methylation and BRCA1 (exon 1) and BRCA2 (exon 2) exon methylation in breast (cancer) cells^15^. Therefore, we also evaluated differential methylation in these specific regions, except for the BRCA1 and BRCA2 exonic regions where coverages were too low to pass the filtering step (see Materials and Methods section). As less putatively methylated regions (20) were present in these loci compared to the full genome-wide approach, adjustment for multiple testing is less conservative. However, still no significant results were found using this approach (minimal FDR: 36.1%; Table 3).

**Table 3:** Previously reported candidate differentially methylated loci upon soy treatment.

ID	Ensembl ID	Gene Symbol	logFC	t-statistic	*P*-value	FDR
40450702	ENSG00000012048	BRCA1	0.601571	2.318641	0.026147	0.361056
41499320	ENSG00000077092	RARB	0.435366	1.918658	0.062905	0.361056
42129392	ENSG00000161055	SCGB3A1	−0.60998	−1.74276	0.089829	0.361056
41919946	ENSG00000164362	TERT	0.460141	1.667295	0.104048	0.361056
39964752	ENSG00000139618	BRCA2	0.35836	1.602313	0.11774	0.361056
40450701	ENSG00000012048	BRCA1	0.471881	1.601995	0.11781	0.361056
42042450	ENSG00000134982	APC	−0.55273	−1.52263	0.136503	0.361056
42129390	ENSG00000161055	SCGB3A1	−0.35985	−1.49163	0.144422	0.361056
40582446	ENSG00000206075	SERPINB5	0.362625	1.414938	0.165595	0.365381
40450700	ENSG00000012048	BRCA1	0.332877	1.358449	0.182691	0.365381
42129393	ENSG00000161055	SCGB3A1	−0.24687	−1.15369	0.256156	0.446046
40582443	ENSG00000206075	SERPINB5	−0.30749	−1.1258	0.267628	0.446046
39692687	ENSG00000084207	GSTP1	0.196571	0.806211	0.425363	0.624811
41919949	ENSG00000164362	TERT	−0.17685	−0.7853	0.437368	0.624811
40582445	ENSG00000206075	SERPINB5	0.099288	0.421192	0.676094	0.835414
42129391	ENSG00000161055	SCGB3A1	−0.08725	−0.38149	0.705056	0.835414
41919948	ENSG00000164362	TERT	−0.10214	−0.37464	0.710102	0.835414
40582447	ENSG00000206075	SERPINB5	−0.06612	−0.29675	0.768347	0.853719
41919950	ENSG00000164362	TERT	−0.02402	−0.1002	0.920733	0.928029
40582444	ENSG00000206075	SERPINB5	−0.02584	−0.09095	0.928029	0.928029

Columns indicate Map of the Human Methylome identifiers, Ensemble id, Gene Symbol, log fold-change (logFC), t-statistic, P-value, and False Discovery Rate (FDR).

Furthermore, similar to the analyses by Qin *et al*.^10^, Spearman correlation between log-cpm values and resp. genistein, total daidizein and total isoflavone concentrations was assessed for these 20 regions in the treatment group (Table 4), again without significant results (likewise for Pearson correlations). For RARB, the exact genomic region tested by Qin *et al*.^10^ was unclear, yet evaluation of other regions in this gene did not yield significant results upon FDR adjustment (data not shown). Finally, for completeness, an additional analysis was performed for all regions in gene promoters (49,378) given the well-described functional relevance of promoter methylation, but also this analysis did not yield significant results (data not shown).

**Table 4:** Spearman correlation (ρ) of methylation degrees (log-cpm) and (a) genistein, (b) daidzein and (c) total isoflavone concentrations.

ID	Ensembl ID	Gene Symbol	Genistein			Daidzein			Total Isoflavone		
			ρ	*P*-value	FDR	ρ	*P*-value	FDR	ρ	*P*-value	FDR
40450702	ENSG00000012048	BRCA1	0.2000	0.5835	0.9457	0.4424	0.2042	0.6184	0.2970	0.4070	0.9608
41919946	ENSG00000164362	TERT	0.0545	0.8916	0.9457	0.4788	0.1661	0.6184	0.1758	0.6320	0.9608
39964752	ENSG00000139618	BRCA2	0.3455	0.3305	0.9457	0.5515	0.1043	0.6184	0.4182	0.2324	0.9608
40450701	ENSG00000012048	BRCA1	0.1394	0.7072	0.9457	0.5273	0.1228	0.6184	0.3333	0.3488	0.9608
40582446	ENSG00000206075	SERPINB5	0.1152	0.7588	0.9457	0.4061	0.2474	0.6184	0.2121	0.5599	0.9608
42129393	ENSG00000161055	SCGB3A1	−0.3212	0.3677	0.9457	−0.5152	0.1328	0.6184	−0.2485	0.4916	0.9608
40582443	ENSG00000206075	SERPINB5	0.3576	0.3128	0.9457	0.4667	0.1782	0.6184	0.3091	0.3871	0.9608
40582445	ENSG00000206075	SERPINB5	−0.2242	0.5367	0.9457	−0.4061	0.2474	0.6184	−0.2727	0.4483	0.9608
41499320	ENSG00000077092	RARB	0.0909	0.8114	0.9457	0.3333	0.3488	0.6783	0.0667	0.8648	0.9608
42129392	ENSG00000161055	SCGB3A1	−0.2606	0.4697	0.9457	−0.2970	0.4070	0.6783	−0.1879	0.6076	0.9608
40582447	ENSG00000206075	SERPINB5	−0.2970	0.4070	0.9457	−0.3091	0.3871	0.6783	−0.3697	0.2956	0.9608
40582444	ENSG00000206075	SERPINB5	−0.0545	0.8916	0.9457	−0.3333	0.3488	0.6783	−0.1030	0.7850	0.9608
40450700	ENSG00000012048	BRCA1	0.0424	0.9186	0.9457	0.2242	0.5367	0.7465	0.1879	0.6076	0.9608
41919949	ENSG00000164362	TERT	0.1515	0.6818	0.9457	0.2121	0.5599	0.7465	0.2242	0.5367	0.9608
42129390	ENSG00000161055	SCGB3A1	−0.0909	0.8114	0.9457	0.2606	0.4697	0.7226	0.0061	1.0000	1.0000
42042450	ENSG00000134982	APC	0.2000	0.5835	0.9457	−0.0909	0.8114	0.9311	0.1030	0.7850	0.9608
41919948	ENSG00000164362	TERT	0.0303	0.9457	0.9457	0.0909	0.8114	0.9311	0.1515	0.6818	0.9608
41919950	ENSG00000164362	TERT	0.2727	0.4483	0.9457	0.0788	0.8380	0.9311	0.1758	0.6320	0.9608
39692687	ENSG00000084207	GSTP1	0.2121	0.5599	0.9457	0.0182	0.9728	1.0000	0.0667	0.8648	0.9608
42129391	ENSG00000161055	SCGB3A1	0.0909	0.8114	0.9457	0.0061	1.0000	1.0000	−0.0061	1.0000	1.0000

Columns represent Map of the Human Methylome identifiers, Ensemble identifiers, Gene Symbol, Spearman correlation (ρ), *P*-value, and False Discovery Rate (FDR) for (a) genistein, (b) daidzein and (c) total isoflavone concentration.

#### Pathway analysis

Using a *P*-value cut-off of 0.0005 (see Materials and Methods), Entrez Identifiers of 177 selected regions were considered for both GO enrichment and KEGG pathway analysis. Using this larger set, top GO enrichment results included vacuoles/lysosomes and voltage gated calcium channel gene sets. KEGG pathway analysis includes involvement of signaling pathways such as mTOR, Oxytocine, MAPK, AGE-RAGE among the top results. However, none of these GO terms or KEGG pathways remained significant enriched upon FDR adjustment (Top 10 results in Tables 5 and 6).

**Table 5:** Top 10 results GO analysis for putatively differentially methylated regions (*P* < 0.0005).

GO Term	Category	Number of genes for GO term	Number of DM genes	*P*-value	FDR
Vacuolar part	CC	702	18	3.25E-06	0.068772
Vacuolar membrane	CC	596	15	2.83E-05	0.291939
Vacuole	CC	1184	22	4.14E-05	0.291939
Voltage-gated calcium channel activity involved in cardiac muscle cell action potential	MF	5	2	0.000514	1
Transcriptional activator activity, RNA polymerase II distal enhancer sequence-specific binding	MF	25	3	0.000763	1
L-type voltage-gated calcium channel complex	CC	6	2	0.000768	1
Coreceptor activity involved in Wnt signaling pathway, planar cell polarity pathway	MF	6	2	0.000768	1
Melanosome organization	BP	26	3	0.000857	1
Pigment granule organization	BP	27	3	0.000959	1
Lysosomal membrane	CC	283	8	0.001081	1

Columns represent Gene Ontology (GO) term, Category (being: CC: Cellular Component, MF: Molecular Function and BP: Biological Process), the number of genes in the gene set, the number of “differentially methylated” genes (loose *P* < 0.0005 cut-off), the *P*-value, and False Discovery Rate (FDR).

**Table 6:** Top 10 results for pathway analysis for putatively differentially methylated regions (*P* < 0.0005).

Pathway	Number of genes in pathway	Number of DM genes	*P*-value	FDR
mTOR signaling pathway	151	6	0.00076536	0.248742101
HTLV-I infection	255	7	0.00239934	0.375649312
Oxytocin signaling pathway	153	5	0.00502011	0.375649312
MAPK signaling pathway	295	7	0.00537556	0.375649312
AGE-RAGE signaling pathway in diabetic complications	99	4	0.00577922	0.375649312
Cholinergic synapse	112	4	0.00889251	0.469555659
Cortisol synthesis and secretion	63	3	0.01080852	0.469555659
Long-term potentiation	67	3	0.0127745	0.469555659
Autophagy - animal	128	4	0.01402011	0.469555659

Columns represent KEGG pathway, the number of genes in a pathway, the number of “differentially methylated” genes (loose *P* < 0.0005 cut-off), the *P*-value, and False Discovery Rate (FDR).

## Discussion

Here, we report the first genome-wide *in vivo* study on the impact of soymilk consumption on the glandular mammary epigenome. The aim of this study, exploratory in nature, was to detect consistent methylation differences that are specific to exposure to isoflavones by soy intake. Previously reported *in vitro* experiments^1^ suggest hypomethylation upon soy exposure, which might confer altered breast cancer risk, as reviewed by^16^. Though this study has several limitations (see below), it did not reveal data supporting the latter to be physiologically relevant. Library size estimates appeared to be somewhat lower in the soymilk group, but these estimates are easily affected by experimental/technical variation and are, therefore, no reliable proxies for global DNA methylation. Moreover, LC-MS analysis of global DNA methylation, association with exposure measures, and MethylCap-seq based Line-1 methylation assessment indicated no differences.

Furthermore, locus-specific analysis revealed no significantly differentially methylated loci. Moreover, no significant results for RARβ2 and CCND2 could be observed, though Qin *et al*. linked hypermethylation of these loci with genistein serum concentrations^10^. The latter study reported dose-specific effects of a longer supplementation phase (one menstrual cycle *vs*. 5 days) in a different study population, treated with different sources of isoflavones (soy supplements *vs*. soymilk). Also other previously reported *in vitro* results^14^ could not be reproduced, which is not so surprising as *in vitro* findings are often difficult to validate *in vivo*. Indeed, this is the first genome-wide *in vivo* study effectively assessing the impact of soymilk on glandular breast tissue, which is the most relevant target in the context of breast health. On the other hand, the affinity of MethylCap-seq depends on the sequence context of the methylated regions, implying that overall coverage (and therefore power) for some loci might have been too limited to validate these known findings^17^. Nevertheless, previous studies by our group using the same methodology (and often for lower sample sizes) did typically lead to significant results, which were often successfully independently validated (e.g. Tomar *et al*.^18^, Van Vlodrop *et al*.^19^), implying that the applied methodology is a very unlikely reason for the general lack of significant findings here.

Yet, as the sample size was relatively small in this exploratory analysis, better powered DNA methylation experiments are required to observe potential small effects. Indeed, though this could also be attributed to imperfect normalization of observed library size differences, the *P*-value distribution of the results suggests that such minor effects may be present. Moreover, we cannot exclude potential impact on breast cancer risk, be it positive or negative. For example, some of the top loci in the general differential methylation analysis, TIAM1, DUSP22 and JAK2 are described to be related to proliferation, survival and invasiveness of breast cancer^20–23^. Also, top KEGG pathway analysis results exhibit some pathways that have a known association to breast and other cancers such as mTOR, Oxytocine, MAPK, AGE-RAGE signaling pathways^24–27^. Yet, since both the differential methylation and pathway analyses yielded no significant results upon FDR correction, it is clear that these findings at most point towards the necessity of larger studies to identify possible minor or subpopulation specific effects and do not support major epigenetic modulation of breast cancer risk (positive or negative) as such.

Next to increasing power by larger sample numbers and a possible impact of duration of soy consumption, also the (variance of the) age of the participants should be considered in larger studies. In the study at hand, both control and treatment populations were very heterogeneous (Table 1), as we aimed to identify general (i.e. age-independent) methylation differences in the adult female population due to soy consumption. However, whereas our analyses indicate no large, age-independent differences in breast methylation, recent literature links the possible positive or negative effect of soy consumption on breast cancer risk in adults to the period or age when soy consumption started^4,28^. Early-life exposure to soy may alter estrogen mediated processes and therefore, alter the effect of genistein, daidzein and other isoflavones, which are estrogen antagonists. Whether this effect is sustained by DNA methylation remains largely unknown^28^. Note that also other sources of heterogeneity (e.g. smoking, contraceptive use, menstrual cycle) present in our study may obscure effects only present in specific subgroups.

In conclusion, in this exploratory analysis, we observed no impact of soymilk consumption on the human mammary gland epigenome. Furthermore, our study could not confirm previously described results of either *in vivo* or *in* vitro studies. Therefore, overall, our exploratory results do not support major general impact of short duration soymilk consumption on breast health through DNA methylation. Yet, we suggest that larger scale research with prolonged exposure and on different time points through female development, menstrual cycles and considering age, is essential for a full understanding of the impact of soy metabolites on (epigenetically regulated) breast health.

## Materials and Methods

### Subjects and treatments

A total of 30 generally healthy Belgian or Dutch women, scheduled for an aesthetic breast reduction, were recruited for this study; 20 of them were included in this epigenomics study. The exclusion criteria were breast cancer, antibiotic treatment within the previous month, and soy allergy. Ethical approval was granted by the Ethics Committee of the Ghent University Hospital (EC UZG 2005/022). The volunteers were fully informed of the aims of the study and gave their written consent. All experiments were performed in accordance with relevant local and national guidelines and regulations.

One batch of commercially available soymilk derived from whole soybeans in 250 mL cartons (Alpro^®^ Soya Drink Nature, Alpro NV, Wevelgem, Belgium) was kindly provided by the manufacturer and analyzed in triplicate at study onset and closure as described by Bolca *et al*.^29^. One portion of soymilk (250 mL) contained 16.98 ± 0.76 mg genistein and 5.40 ± 0.22 mg daidzein aglycone equivalents, and 8.25 g proteins, 7 g carbohydrates, 4.75 g lipids, 1.5 g fibers, 0.375 µg vitamin B12, 0.6 µg vitamin B2, and minerals.

### Study design

This study was a randomized dietary intervention trial with a run-in phase of at least 4 days and a supplementation phase of 5 days before breast surgery. Following eligibility assessment, volunteers were randomly allocated to the soymilk (*n* = 10) or control (*n* = 20, of which 10 included in the epigenomics study) group. All participants were asked to abstain from soy-based products during the whole experimental period. A detailed list of isoflavone-containing foods and dietary supplements was distributed in order to guide the volunteers in this respect. Additionally, subjects were instructed to report every case of doubt or fortuitous consumption and to provide detailed information on that eating occasion, including type and portion size. During the supplementation phase, 250 mL soymilk was consumed daily with breakfast, lunch, and dinner. The control group did not receive any supplementation before surgery. Compliance was evaluated by subject inquiry and urinary isoflavone excretion.

Subjects delivered a spot urine sample after the run-in phase and before anesthesia. During surgery (12–18 h after last soy supplementation), blood and breast biopsies were collected. Serum was obtained by centrifugation (10 min at 600 *g*, room temperature) after coagulation. Aliquots of both urine and serum samples were stored at −20 °C until analysis. The tissue samples were immediately frozen in liquid nitrogen and stored at −80 °C until analysis. Without thawing the tissue samples, fractions containing almost exclusively glandular tissue were dissected, based on gross inspection. Areas of adipose tissue intimately intermixed with fibroglandular tissue were avoided and connective tissue was removed. Before processing, all samples were randomized and the investigators were blinded to the treatments when working with the samples.

In addition, a general questionnaire was used to obtain information on the subjects’ history of antibiotic treatments, hormonal therapies, other medication, food supplement intakes, and anthropometric measures. Each participant reported her habitual fat and fiber intakes, and her soy consumption since birth using validated self-administered food-frequency questionnaires^30^.

### Chemicals

Genistein, daidzein, and equol were purchased from Extrasynthèse (Genay, France), and dihydrodaidzein and *O*-desmethylangolensin from Plantech UK (Reading, UK). For the hydrolysis of conjugated isoflavones, a 33 g/L-solution of Type H-1 *Helix pomatia* extract (min. 300 U β-glucuronidase/mg and 15.3 U sulfatase/mg; Sigma-Aldrich, Bornem, Belgium) in sodium acetate buffer (0.1 mol/L, pH = 5) was prepared. A 400 µmol/L and 40 µmol/L-solution of 4-hydroxybenzophenone in methanol was used as internal standard in the quantitative analyses of urine, serum, and breast tissue, respectively.

### Exposure to isoflavones and 17β-estradiol

Genistein, daidzein, dihydrodaidzein, equol, and *O*-desmethylangolensin in urine were quantified upon enzymatic hydrolysis and liquid-liquid extraction, using a LC-MS method validated by Wyns *et al*.^31^. Based on a creatinine clearance rate of 0.163 mmol/(d.kg)^32^, daily urinary isoflavone excretions were calculated^33^. Quantification of genistein, daidzein, dihydrodaidzein, equol, *O*-desmethylangolensin, and the internal standard in hydrolyzed serum (200 µL) and glandular tissue (250 mg) was performed by LC-MS/MS according to Bolca *et al*.^5^.

Additionally, estrogens were extracted from glandular breast tissue (200 mg) as described by Chetrite *et al*.^34^ and analyzed for 17β-estradiol using a quantitative immunoassay (EIA-4499, DRG Instruments GmbH, Marburg, Germany).

### MethylCap-seq

High-quality genomic DNA (gDNA) (20–50 kb; 11.04–127.42 ng/mg) was obtained from 22 mammary gland samples (10 soymilk, 10 matched controls, 2 technical replicates – one for each treatment group) using the PureLink Genomic DNA Mini kit (Invitrogen, Merelbeke, Belgium). Briefly, 50–85 mg of manually dissected glandular tissue was lysed with proteinase K (20 g/L) for 4 h at 55 °C and 300 rpm. The lysate was centrifuged (3 min at 17968 *g*, room temperature) and treated with RNase A (20 g/L in 50 mM Tris-HCl (pH 8.0), 10 mM EDTA; 2 min at room temperature). The sample was purified through a silica-based membrane and gDNA was eluted twice with 50 µL of water. DNA quantity and fragment length were measured using Qubit dsDNA HS assay (Invitrogen, Merelbeke, Belgium) and 1% agarose gelelectrophoresis, respectively, and all samples were diluted to a final concentration of 500 ng gDNA/75 µL water.

MethylCap-seq is a genome-wide DNA methylation profiling methodology combining methyl binding domain (MBD) based capture of methylated fragments and subsequent massive-parallel sequencing, also known as MBD-seq^35–37^. MethylCap-seq was performed by NXTGNT (Ghent, Belgium) as outlined earlier^38^, with following modifications: (a) 500 ng input material was used for the affinity purification step (MethylCap kit, Diagenode, Liege, Belgium), (b) paired-end (2 × 51 nt, excluding adapters and multiplex identifiers) massive parallel sequencing was performed on the Illumina HiSeq2000 platform.

### Global DNA methylation percentages

Global DNA methylation levels were assessed as previously reported^39^. As a minimum amount of 1 µg gDNA input was required, analyses were performed on the qualifying subset of 6 treatment and 5 control samples. In summary, gDNA was enzymatically hydrolyzed to deoxyribonucleosides and dissolved in LC-grade water. Similarly, stock solutions were prepared for reference standards of 2′-deoxycytidine (dC) and 5-methyl-2′-deoxycytidine (5-mdC), purchased from Sigma (D3897-1G) and Jena Bioscience (N-1044) respectively, to create a series of calibration standards, used in all of the experiments. Global DNA methylation was assessed by quantifying both 5-mdC and dC using Waters Acquity UPLC coupled to Waters Micromass Quattro Premier Mass Spectrometer (electrospray ionization in positive mode). Global DNA methylation was calculated as a percentage of 5-mdC versus the sum of 5-mdC and dC.

### Data analysis

For MethylCap-seq, sequence reads were mapped to the human reference genome (GRCh37) with BOWTIE^40^ and aligned fragments were summarized using the in-house developed Map of the Human Methylome (http://www.biobix.be/map-of-the-human-methylome/). As previously described in e.g. Van Vlodrop *et al*.^19^, this map contains genomic regions that are putatively independently methylated, without relying on existing gene annotation. For each of these regions, the maximum number of mapped fragments (“peak height”) per sample was used for subsequent analysis (duplicate fragments removed). For annotation, we relied on Ensembl Genes 91. Unless mentioned otherwise, subsequent data analysis was performed using R (3.3.2)^41^.

For the exploratory analysis, the technical replicates were included and log-transformed counts per millions (log-cpm) values were calculated using the “cpm” function present in the R Bioconductor EdgeR package (version 3.16.5)^42^. Subsequently, the 10,000 loci with most variance were selected for hierarchical clustering (“complete” method on Euclidean distance matrix, using the “hclust” function).

For the statistical assessment of differential methylation, those technical replicates with the lowest overall coverage where removed from the analysis. Average log-cpm values were used to compare the genic-intergenic resp. promoter-genic methylation ratios between treatment groups. For the locus specific comparisons, only those loci featured by Gene Symbol annotation and an average coverage of at least 4 (prior to normalization) were used. This filtering step, aiming to remove uninformative variables from the data set, together with the removal of duplicate entries led to a final set of 759,275 loci out of a total of 3,618,706.

For the analysis of differentially methylated loci, the limma-voom model was used^43^, since limma-voom presented considerably improved quality statistics compared to others (based on MA-plot and *P*-value distribution, data not shown). As is standard practice for limma-voom, TMM normalization (from EdgeR package) was performed on the filtered counts^42,44^. Known to be often associated with gene expression, particularly promoter methylation is of interest, leading us to focus on the 49,378 promoter regions (2000 bp upstream to 500 bp downstream of canonical transcription start site, based on Ensembl annotation) for the promotor specific differential methylation analysis.

Gene Ontology (GO) enrichment analysis was performed using the “goana” function in the limma package. This package uses Entrez gene identifiers and can simultaneously report on the three main GO categories: biological processes, molecular functions and cellular components. For gene set analysis, a *P*-value cutoff (*P*-value < 0.0005) was used rather than a multiple testing adjusted cut-off given the general lack of significant results for the individual genes (see Results). Although individual loci considered are thus less reliable, this is putatively compensated by the higher number of actually differentially methylated loci, leading to increased power for the gene set analysis. Additionally, pathway analysis was investigated using the “kegga” function in the limma package. Cut-off criteria and *P*-value correction were performed analogously to the GO enrichment analysis.

LINE-1 methylation was evaluated by mapping (BOWTIE, loose parameter settings: L = 5, e = 100) both paired-end reads separately on the Homo Sapiens LINE-1 consensus sequence (L1HS) obtained from Repbase^45^ and counting the mapped reads. Duplicate reads were removed first using FastUniq^46^. Paired-ends mapping both to L1HS were counted as a single event. L1HS count data were normalized for coverage differences by division by the total number of sequenced fragments.

Additional data analyses were performed in R (3.3.2) as well: the Student *t*-test was used to compare sequencing characteristics, LINE-1 methylation, and global DNA methylation between groups, the Wilcoxon rank-sum test for the comparison of 17β-estradiol concentration and both genic-intergenic and promoter-genic methylation ratios between treatment groups, and Spearman correlation to evaluate the correlation between isoflavone concentrations and library sizes resp. locus-specific methylation degrees. Throughout the manuscript, Benjamin-Hochberg *P*-value adjustment was performed to correct for multiple testing, leading to False Discovery Rate (FDR) estimates.

## Data Availability

The datasets generated and analysed during the current study are available in the Gene Expression Omnibus (GEO) repository, GSE112727.
